# Physical Performance Profiles of Highly Trained U14–U19 Youth Soccer Players: Age-Category Differences and Associations with Maturity Offset

**DOI:** 10.3390/sports14090402

**Published:** 2026-09-14

**Authors:** Krisztián Havanecz, Ferenc Ihász

**Affiliations:** 1 Training Theory and Methodology Research Center, Hungarian University of Sports Science, 1123 Budapest, Hungary; 2 Department of Health Promotion and Exercise Science, Széchenyi István University, 9026 Győr, Hungary; ihasz.ferenc@sze.hu

**Keywords:** anthropometry, biological maturation, body composition, peak height velocity, physical performance, youth soccer

## Abstract

This study aimed to examine age- and maturity-related differences in anthropometric characteristics, body composition, functional movement, and field-based physical performance among U14–U19 male youth soccer players. Seventy academy players completed anthropometric and body composition assessments, the Functional Movement Screen, a 30-m linear sprint, the Illinois Agility Test with and without the ball to assess change-of-direction speed (CODs), a standing long jump, and the Yo-Yo Intermittent Recovery Test Level 1. Biological maturation was estimated using the Mirwald equation. Age-category differences were examined using one-way ANOVA or Welch’s ANOVA, as appropriate. Associations between variables were assessed using Pearson’s correlation coefficient, while multivariable linear regression models were used to examine the independent associations of chronological age and maturity offset with physical performance outcomes. Significant age-category differences were observed in estimated age at peak height velocity (F(4, 65) = 5.12, η^2^ = 0.240), maturity offset (F(4, 65) = 45.12, η^2^ = 0.735), body height (F(4, 32.15) = 5.01, η^2^ = 0.319), body weight (F(4, 65) = 15.47, η^2^ = 0.488), skeletal muscle mass (F(4, 65) = 16.36, η^2^ = 0.502), 30-m sprint time (F(4, 65) = 27.97, η^2^ = 0.633), CODs without a ball (F(4, 65) = 16.52, η^2^ = 0.504), standing long jump (F(4, 65) = 11.39, η^2^ = 0.412), and endurance test performance (F(4, 30.21) = 16.47, η^2^ = 0.408; all *p* < 0.01). The U14 players generally demonstrated less favorable performance than the older age categories. Interestingly, no significant age-category differences were observed in body fat percentage, Functional Movement Screen total score, or CODs with the ball. In the regression analyses, maturity offset was significantly associated only with standing long jump performance (β = 0.496, *p* = 0.012), but the association did not remain significant after Holm correction. The reported age-category values may provide preliminary benchmarks for evaluating youth academy soccer players, but they should be interpreted alongside maturity status and individual developmental characteristics. Future studies could complement field-based testing with biomechanical and treadmill-based laboratory assessments to provide a more comprehensive evaluation of age- and maturity-related performance.

## 1. Introduction

Soccer performance is determined by multiple dimensions and the interaction of technical, tactical, psychological, and physical qualities. From a physical perspective, match play is characterized by intermittent activity patterns, including repeated accelerations, decelerations, change in direction, short sprints, jumps, and periods of short recovery [1,2]. These demands have been extensively described in adult soccer, while increasing attention has also been given to youth and academy players. Vieira et al. summarized match running performance in young soccer players, whereas Harper et al. highlighted the relevance of high-intensity acceleration and deceleration demands in team-sport match play [3,4]. In academy environments, Morgans et al. also showed that physical match demands differ between youth and senior players, supporting the need to interpret youth players’ physical profiles within their own developmental context [5].

In recent years, wearable tracking technologies such as global positioning system/global navigation satellite systems have become widely used to quantify external load during soccer training and match play [6]. These systems provide valuable information about what players actually perform in applied settings, including total distance, high-speed running, sprinting, accelerations and decelerations [7]. However, match- and training-derived data explain only a fragment of the physical capacities of the players. Therefore, field-based and laboratory-based test batteries remain important tools in academy settings, because they allow practitioners to assess specific physical qualities under standardized conditions. Paul and Nassis emphasized that physical fitness testing in youth soccer should be interpreted with careful attention to reliability, validity, and sensitivity, while Dugdale et al. provided support for the use of several common field-based fitness tests in youth soccer players [8,9].

In practice, these test batteries often include anthropometric and body composition assessment, functional movement screening, linear sprinting, change-of-direction speed (CODs), agility, lower-body power, and intermittent endurance tests in the preparatory phase of a macrocycle. Sprint tests are commonly used to assess acceleration and maximal running speed, jump tests provide information about lower-body explosive power, and agility or CODs tests are used to evaluate the ability to rapidly reorganize movement in response to task demands. CODs primarily reflects the physical ability to alter movement direction without a reactive component, whereas agility involves a rapid whole-body change in velocity or direction in response to a stimulus [10]. The Yo-Yo Intermittent Recovery Test Level 1 (Yo-Yo IRTL1) is also frequently applied in soccer because it reflects the intermittent nature of the game; Deprez et al. reported reliability and validity data for this test in young soccer players [11]. Functional movement assessments may provide additional information about fundamental movement quality, although their role should not be interpreted as direct measures of functional capacity or soccer performance. In young soccer players, Lloyd et al. examined the relationships between functional movement screen scores, maturation, and physical performance, showing that movement quality and performance-related variables should not be interpreted as identical constructs [12].

The interpretation of these results is especially challenging during adolescence. Players within the same chronological age group may differ substantially in biological maturity, body size, body composition, neuromuscular development, and functional capacity. This is highly relevant in youth soccer because players within the same chronological age category can be pre-, circa-, or post-peak height velocity (PHV). Mirwald et al. proposed an anthropometric method to estimate maturity offset, while Philippaerts et al. demonstrated that physical performance in youth soccer players changes markedly around PHV [13,14]. Consequently, chronological age alone may be insufficient to explain differences in sprinting, jumping, agility, or endurance performance among academy soccer players. Accordingly, it may conflate age-related differences with variation in biological maturation, thus favoring earlier-maturing players and underestimating the capacities of their later-maturing peers.

Previous studies have consistently shown that biological maturation influences the physical profile of young soccer players. Malina et al. (2004) reported maturity-associated variation in the growth and functional capacities of youth soccer players aged 13–15 years [15]. Similarly, maturity status was related to differences in body size, functional capacity, soccer-specific skills, and goal orientation in players aged 11–14 years [16]. Deprez et al. further showed that relative age and biological maturation were associated with anthropometric and anaerobic characteristics in elite youth soccer players [17]. These findings indicate that early maturing players may have temporary advantages in speed-, strength-, and endurance-related tasks, which can influence how coaches and practitioners evaluate player potential.

At the same time, maturation does not necessarily influence all performance domains to the same extent. Previously it has been reported that biological maturation affected morphology and fitness more strongly than motor coordination in 15–16-year-old elite youth soccer players [18]. This distinction is important from an applied perspective because linear sprinting, jumping, and intermittent endurance may be more directly affected by body size, muscle mass, and neuromuscular development, whereas agility and coordination are influenced by a more complex interaction of physical, technical, and perceptual factors. More recent studies have further supported this interpretation by showing that biological maturity was associated with several physical performance outcomes in elite youth soccer players [19]. However, another study reported that maturity had a stronger association with sprint performance than relative age in academy soccer players [20].

These findings are also consistent with contemporary youth development models. The well-known long-term athlete development approaches emphasize the progressive development of physical qualities across childhood and adolescence [21]. However, the youth physical development model proposed by Lloyd and Oliver provides a more maturation-sensitive framework for both male and female athletes, suggesting that training and performance evaluation should not be based only on chronological age [22]. In line with this, the international consensus on youth resistance training also highlighted that exercise programming for young athletes should consider biological maturation, training history, technical competency, and individual readiness rather than age alone [23]. From a practical standpoint, this means that football academy practitioners should interpret test results in relation to both chronological age and maturity status, particularly when comparing players across age groups or identifying individual strengths and weaknesses.

Although previous studies have examined age- and maturity-related differences in youth soccer players, several limitations remain. Many investigations have focused on relatively narrow age ranges, selected performance outcomes, or isolated components of the player profile. Fewer studies have examined a broad academy pathway using a multidimensional test battery that integrates anthropometry, body composition, somatic maturation, functional movement, sprint performance, CODs with and without the ball, lower-body power, and intermittent endurance. Examining these components simultaneously across the U14–U19 academy pathway under standardized testing conditions may provide a more comprehensive view of age- and maturity-related differences within the same academy environment.

Therefore, the aim of this study was to examine age-category differences in anthropometric, body composition, functional movement, and field-based physical performance characteristics among highly trained male youth soccer players from an academy setting. It was hypothesized that players in older age categories would demonstrate superior sprint, jump, change-of-direction, and intermittent performance, whereas differences in functional movement quality and CODs with the ball would be less pronounced. A secondary aim was to explore the associations between these outcomes.

## 2. Materials and Methods

### 2.1. Study Design

This study used a cross-sectional observational design to examine age- and maturity-related differences in anthropometric, body composition, functional movement, and field-based physical performance characteristics among academy male soccer players. All assessments were conducted during the first week of the pre-season and formed part of the academy’s regular performance profiling procedure. Players entered the testing period after a one-month off-season period without structured team training or matches. During the two weeks preceding the assessments, players from the U15 age category and above were instructed to complete a home-based program three times per week, with each session lasting no longer than 60 min and focusing on general strength development and aerobic capacity. The testing battery was completed over two consecutive days. On the first day, anthropometric and body composition measurements were performed followed by a functional movement test. On the second day, players completed the field-based physical performance tests. Players from U14, U15, U16, U17, and U19 age categories were included. Before testing, all players received standardized information about the aims and procedures of the assessments, with particular attention given to the U14 players because they had no previous experience with this type of testing battery. Therefore, before data collection, they completed familiarization trials for each physical performance test to ensure that they understood the procedures and could perform them correctly. All tests were administered by the medical and strength and conditioning staff under standardized academy conditions.

### 2.2. Participants

Initially, 134 male academy players from the U14 to U19 age categories were considered for inclusion in the study. No separate U18 group was included because the national youth competition structure did not comprise a distinct U18 age category. Players were eligible if they were registered members of the academy and completed the full pre-season test battery required for the present analysis. A complete-case approach was applied; players with missing data for one or more primary study variables, or those unable to complete the assessments because of injury, illness, or absence, were excluded. Accordingly, 64 of the 134 potentially eligible players (47.8%) were excluded; the specific reasons for noncompletion were not recorded separately. The final sample therefore consisted of 70 players (U14: *n* = 16; U15: *n* = 11; U16: *n* = 14; U17: *n* = 14, U19: *n* = 15). The players represented academy teams competing at the highest national youth level in their respective age categories. The typical weekly training schedule comprised four soccer sessions for the U14–U16 players and five for the U17–U19 players, supplemented by two strength and conditioning sessions for all age groups. The final sample had a mean chronological age of 15.62 ± 1.58 years and a mean estimated age at PHV of 14.82 ± 0.78 years. The mean maturity offset relative to PHV was +0.81 ± 1.37 years. Mean body height and total body weight were 175.86 ± 7.96 cm and 64.12 ± 10.81 kg, respectively.

The study was conducted in accordance with the Declaration of Helsinki and was approved by the Ethics Committee of the Hungarian University of Sports Science (MTSE-KEB/No07/2025). Before data collection, all players and their parents or legal guardians were informed about the aims, procedures, and voluntary nature of the study. Written informed consent was obtained before participation.

### 2.3. Tests and Measurements

All tests are described in the order in which they were conducted.

Anthropometric measurements were performed by a physiotherapist with International Society for the Advancement of Kinanthropometry Level 2 accreditation. Measurements were conducted using standard anthropometric procedures and equipment, including a skin marker, anthropometric measuring devices and tape, and skinfold calipers [24]. The anthropometric assessment included body height (cm) and maturity-related variables. Body composition was assessed using bioelectrical impedance analysis with an InBody 770 device (InBody Co., Seoul, Republic of Korea). The assessment was performed under standardized conditions: participants were instructed to arrive fasting, refrain from fluid intake, and no supervised training session was conducted during the 12 h preceding the bioelectrical impedance assessment. The following body composition variables were retained for analysis: skeletal muscle mass (kg), and body fat percentage (%). Somatic maturity was estimated from anthropometric variables using the sex-specific Mirwald equation for boys [13]. Maturity offset was calculated as:

Maturity offset (years) = −9.236 + [0.0002708 × (leg length × sitting height)] − [0.001663 × (chronological age × leg length)] + [0.007216 × (chronological age × sitting height)] + [0.02292 × ((body mass/body height) × 100)].

Estimated age at PHV was calculated by subtracting maturity offset from chronological age and was used to describe the estimated timing of PHV. Positive maturity offset values indicated that the player was estimated to be post-PHV, whereas negative values indicated that the player was estimated to be pre-PHV [13].

Functional movement quality was assessed using the Functional Movement Screen (FMS) [25,26]. The test battery included the deep squat, hurdle step (left and right leg), in-line lunge (left and right leg), shoulder mobility (left and right arm), active straight-leg raise (left and right leg), trunk stability push-up, and rotary stability tests. Each movement task was scored according to the standard FMS criteria (0–3), where higher scores indicate better movement quality [27]. The total FMS score was calculated as the sum of the individual test scores. For unilateral tests, both sides were assessed, and the lower score was used for the composite score.

Before field-based physical performance tests, players completed a standardized warm-up consisting of mobility work, general running drills without the ball and progressive runs. All field tests were performed on the artificial turf surface on which players regularly trained. The tests were completed in the following order: standing long jump, 30-m linear sprint, Illinois Agility with and without the ball, and Yo-Yo IRTL1. Each test was performed twice, except for the endurance test, and the best valid attempt was retained for analysis. Players were allowed ~1–2 min of recovery between repeated attempts and 3–5 min before proceeding to the next test. Linear sprint was assessed with 30-m using tripod-mounted photocells (Witty Wireless Training Timer, Microgate, Bolzano, Italy). Sprint times were recorded in seconds, with lower values indicating better performance. CODs was assessed using the Illinois Agility Test with and without the ball [28]. Both change-of-direction tests were performed on the same course configuration and in the same direction, with players moving from left to right. Lower-body explosive power was assessed using the standing long jump test, which is commonly used in youth fitness assessment batteries [29]. Jump distance was measured in centimeters using a measuring tape. Endurance capacity was assessed using the Yo-Yo IRTL1 [11]. Total distance covered during the test was recorded in meters and used as the primary intermittent endurance outcome.

### 2.4. Statistical Analysis

All statistical analyses were performed using JASP software, version 0.97. Normality was assessed using the Shapiro–Wilk test and visual inspection of Q–Q plots. Descriptive statistics are presented as mean ± SD. Differences between age categories were examined using one-way ANOVA. Homogeneity of variance was assessed using Levene’s test. When the assumption of homogeneity was met, Tukey’s post hoc test was used; when this assumption was violated, Welch’s ANOVA and Games–Howell post hoc comparisons were applied. Effect sizes were reported using eta squared (η^2^). Associations between test battery variable outcomes were examined using Pearson’s correlation coefficient. Multivariable linear regression models were constructed for each primary physical performance outcome, with chronological age and maturity offset as predictors. Unstandardized regression coefficients with 95% CIs, standardized regression coefficients (β), *p*-values, model R^2^, and variance inflation factors (VIF) were reported. To account for multiple testing, Holm’s correction was applied to the maturity-offset *p*-values from the six regression models. The level of statistical significance was set at *p* < 0.05. Because all eligible complete cases were included, no a priori sample-size calculation was performed. With five groups and n = 70, sensitivity analysis indicated 80% power (α = 0.05) to detect an effect of f = 0.43 (η^2^ = 0.155).

## 3. Results

Descriptive data for each age category are presented in Table 1.

Estimated age at PHV differed significantly between age categories, F(4, 65) = 5.12, *p* = 0.001, η^2^ = 0.240. Tukey post hoc comparisons showed that estimated age at PHV was higher in U17 than in U14 players and higher in U19 than in U14 and U15 players. Maturity offset also differed significantly between age categories, F(4, 65) = 45.12, *p* < 0.001, η^2^ = 0.735. For U14 players, they had lower values than all older age categories, while U19 players had the highest score. Significant age-category differences were also found for body height, body weight, and skeletal muscle mass (*p* < 0.01). For body weight and skeletal muscle mass, U14 players had lower values compared to older age categories. Body fat percentage did not differ significantly between age categories. For field-based physical performance variables, significant age-category differences were found for 30-m sprint time, CODs without the ball, standing long jump, and Yo-Yo IRTL1 performance (all *p* < 0.001). U14 players performed worse than all older groups in the first three tests. Compared with U19 players, they were 0.59 s slower in the sprint (95% CI [0.42, 0.77]), 1.05 s slower in the CODs without the ball (95% CI [0.65, 1.45]) and jumped 35.41 cm less (95% CI [18.39, 52.42]). In the Yo-Yo IRTL1, U14 players covered less distance than U16, U17, and U19 players, and 762 m less than U19 players (95% CI [312, 1212]). No significant differences were found for CODs with the ball (Table 2).

**Figure 1:**
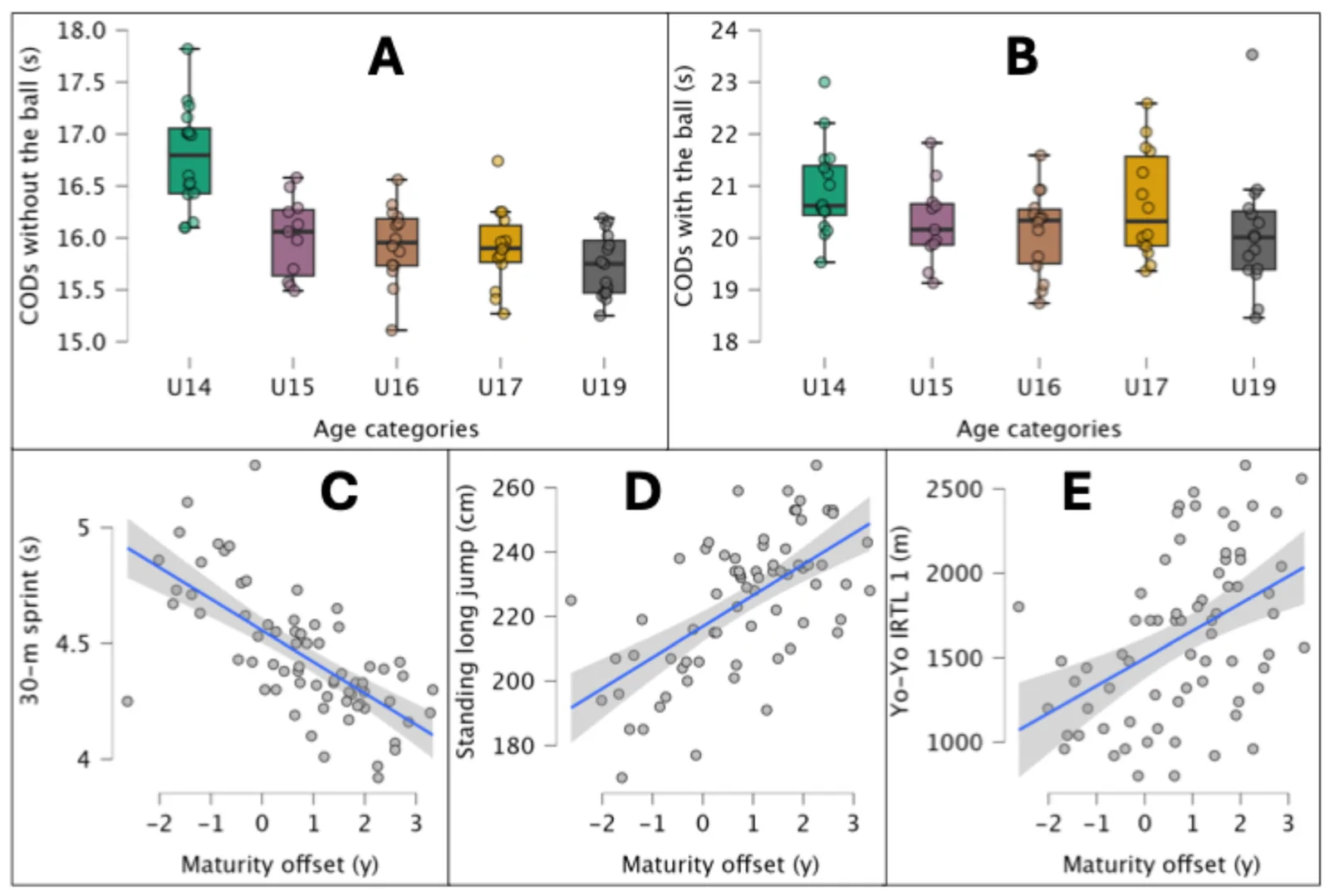
Age- and maturity-related patterns in selected field-based performance outcomes: (**A**) CODs without the ball and (**B**) CODs with the ball across age categories; (**C**) 30-m sprint time, (**D**) standing long jump, and (**E**) Yo-Yo IRTL1 performance according to maturity offset. Points represent individual player observations. Lines and shaded areas in panels (**C**–**E**) represent unadjusted linear regression fits and 95% CIs, respectively. Abbreviations: CODs = change-of-direction speed; Yo-Yo IRTL1 = Yo-Yo Intermittent Recovery Test Level 1.

Figure 1 illustrates change-of-direction performance across age categories and the associations of maturity offset with 30-m sprint, standing long jump, and Yo-Yo IRTL1 performance.

**Figure 2:**
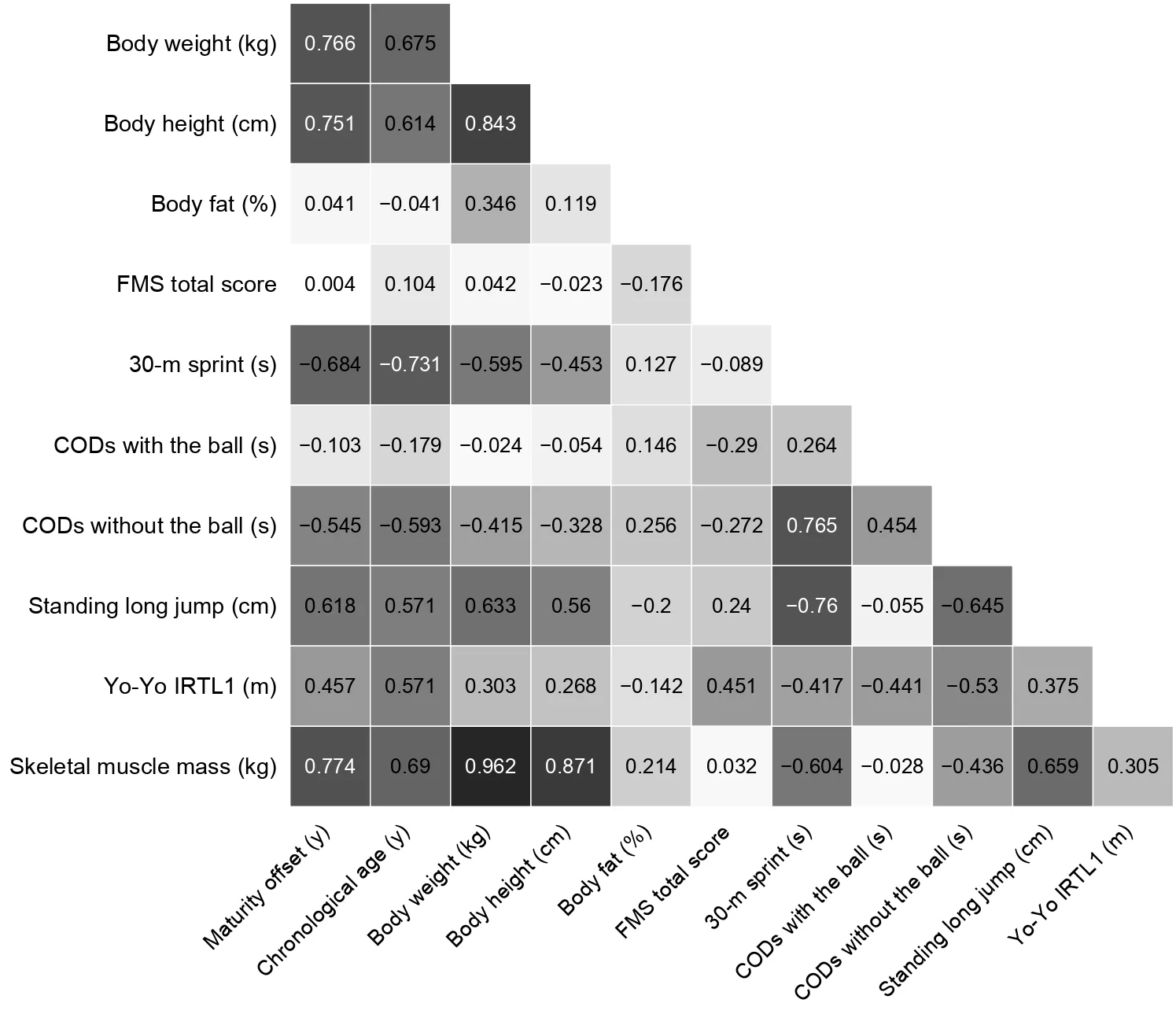
Correlation matrix showing the relationships among maturity-related, anthropometric, body composition, functional movement, and field-based physical performance variables. Darker shading indicates stronger absolute correlation coefficients, while the sign of *r* indicates the direction of the association. Abbreviations: PHV = peak height velocity; FMS = Functional Movement Screen; Yo-Yo IRTL1 = Yo-Yo Intermittent Recovery Test Level 1; CODs = change-of-direction speed.

Figure 2 provides an overview of the bivariate associations observed among the study variables.

In the regression models, maturity offset was independently associated only with standing long jump performance (β = 0.496, *p* = 0.012). However, this result did not remain significant after Holm correction (adjusted *p* = 0.074; Table 3).

## 4. Discussion

The present study identified substantial age-category differences in anthropometric characteristics and several physical performance outcomes among highly trained U14–U19 soccer players. These findings should be considered within the multidimensional nature of soccer performance, which also depends on technical and cognitive abilities [30].

The significant differences observed in estimated age at PHV and in maturity offset highlight that chronological age alone is not the sole variable for assessing players’ physical abilities and stage of biological development. The large effect size of maturity offset (η^2^ = 0.735) indicates clear age-category differences in the players’ estimated position relative to PHV. However, in the multivariable analyses, maturity offset was independently associated only with standing long jump performance, although this association did not remain significant after Holm correction. Overall, the results provide limited evidence for associations between maturity offset and physical performance independent of chronological age.

It is worth noting that the rate of change in body size does not necessarily explain the rate of maturation in individuals, since in this study we considered only single measurements. The lack of differences in body fat (%) across age groups is not necessarily expected, since previous findings suggest that elite athletes of similar ages may be leaner than their sub-elite counterparts [31]. We believe that these differences may be partly the result of country-specific variations in this obesity epidemic.

Significant age-category differences were observed in body height, body weight, and skeletal muscle mass. The younger age groups, particularly the U14 players, consistently exhibited lower values for body height and skeletal muscle mass than older age groups. These anthropometric differences may contribute to better performance on physical tests among more mature players [32]. Interestingly, body fat percentage did not show a significant difference across age groups, which may indicate that the increase in lean body weight was largely related to the maturation process, whereas relative fat content varied less across the U14–U19 age categories.

Body fat percentage decreases with increasing chronological age among adolescents [33], which may support physical performance, running economy, and the ability to change direction. In young athletes, a higher-than-normal body fat percentage content can impair muscle function and mobility, making it difficult to sustain high-intensity activities during soccer matches [34].

Interestingly, no significant age-category differences were found in FMS total score. This suggests that movement quality, as reflected by the composite FMS score, may vary less across adolescent age groups than sprint, jump, or endurance performance in trained youth soccer players [12].

The U14 players were significantly slower than the older age groups in the 30-m sprint and the CODs test without the ball, and they achieved shorter distances in the standing long jump. These differences may partly reflect age- and maturation-related changes in muscle morphology and stretch-shortening cycle function during adolescence [35,36]. In youth soccer, maturity status has also been associated with repeated-sprint performance and other physical fitness outcomes [37]. Similarly, the shorter distance covered by the younger age categories in the Yo-Yo IRTL1 may reflect lower intermittent endurance capacity at this stage of development [14,37]. However, given the cross-sectional design, these between-group differences should not be interpreted as direct evidence of individual developmental changes.

Interestingly, no significant differences were observed between age groups in the CODs test with the ball. This may suggest that performance in this task may depend not only on physical capacities but also on ball-control skills and movement coordination developed through soccer-specific practice. From an applied perspective, this may indicate that ball-related CODs is less closely aligned with age- and maturation-related physical differences than CODs without the ball. However, because the Illinois Agility Test follows a predetermined movement path and does not include a reactive stimulus, these results should not be interpreted as reflecting agility or perceptual-cognitive performance. The present findings are more appropriately interpreted in terms of change-of-direction performance. Previous research has examined the relationships between linear sprint, lower-body power, and change-of-direction performance in soccer players [38]. Age-category differences in change-of-direction, sprint, and jump performance have also been reported [39]. Therefore, the poorer performance observed in the younger age groups without the ball may primarily reflect age- and maturation-related differences in physical and neuromuscular characteristics.

Overall, the findings indicate substantial differences in physical performance across chronological age categories, whereas the independent association of maturity offset with performance was limited to standing long jump. These results highlight the importance of considering chronological age and estimated maturity as related, rather than independent, characteristics when evaluating youth players. Future studies are encouraged to explore modifications to training program interventions that could reduce performance differences arising from maturity differences across age groups or from the interaction between maturity status and the athlete’s long-term developmental outcomes.

## 5. Limitations

The present findings should be interpreted with several limitations in mind. The study was based on a single pre-season testing period and used a cross-sectional design. Therefore, the results describe differences between age groups at one point in time but do not show how individual players develop over time. These differences may also reflect accumulated training experience or cohort-specific characteristics, which cannot be separated from maturation-related influences in the present study. A further limitation is that only the U15–U19 players were assigned a home-based program before testing, resulting in unequal pre-test preparation that may have influenced between-group comparisons. Although U14 players completed familiarization trials, their lack of previous experience with the test battery may have influenced between-group comparisons. All participants were male and belonged to the same football academy system. This, however, ensured similar testing conditions and a relatively homogeneous training environment; nevertheless, the findings may not be applicable to players from other football academies, competitive levels, or female players. In addition, restricting the analysis to complete cases reduced the sample from 134 to 70 players and may have introduced selection bias. Biological maturation was estimated using the Mirwald equation, which is subject to individual prediction error and potential age-related bias, particularly in early- and late-maturing individuals [40]. Given these limitations and the risk of maturity-status misclassification [41], maturity offset was retained as a continuous variable rather than used to classify players as pre-, circa-, or post-PHV. Furthermore, body composition was assessed in the morning, but the testing room was not air-conditioned. Variations in ambient temperature may have influenced the measurement conditions, while bioelectrical impedance estimates may also have been affected by hydration status. Although the test battery covered several important areas of physical performance, it did not contain countermovement or squat jump assessments, which could have provided additional information on lower-limb power and neuromuscular performance.

## 6. Practical Applications

The sample-specific age-category values may provide preliminary benchmarks for identifying expected performance levels and future development targets. However, individual test results should also be interpreted in relation to maturity status and previous testing outcomes, particularly when comparing players of similar chronological age. Considering these physical outcomes alongside technical and tactical assessments may help avoid overemphasizing potentially temporary maturity-related advantages. Repeated multidimensional testing may therefore help practitioners monitor individual development and inform training decisions by identifying specific areas requiring further attention.

## 7. Conclusions

This study highlights that, overall, the physical performance profiles differ across age categories in male youth academy soccer players, although these differences are not consistent across all performance domains. After accounting for chronological age, maturity offset was not independently associated with any physical performance outcome. Age-group differences were primarily evident in anthropometric characteristics, skeletal muscle mass, and sprint, jump, and endurance performance, whereas there were fewer differences in functional movement and CODs with the ball. These findings may provide useful age-category benchmarks for practitioners, provided that they are used within repeated, multidimensional assessments and interpreted alongside biological maturity and individual developmental differences.

## Figures and Tables

**Table 1:** Descriptive characteristics of the study variables by age category.

Variables	U14 (*n* = 16)	U15 (*n* = 11)	U16 (*n* = 14)	U17 (*n* = 14)	U19 (*n* = 15)
Chronological age (y)	13.43 ± 0.33	14.77 ± 0.24	15.63 ± 0.29	16.59 ± 0.29	17.68 ± 0.64
Estimated age at PHV (y)	14.42 ± 0.57	14.50 ± 0.36	14.61 ± 0.33	14.85 ± 0.47	15.34 ± 0.46
Maturity offset (y)	−0.98 ± 0.63	0.26 ± 0.36	1.01 ± 0.38	1.42 ± 1.25	2.34 ± 0.59
Body height (cm)	168.62 ± 9.64	174.91 ± 4.64	177.81 ± 4.74	176.91 ± 5.83	181.49 ± 6.72
Body weight (kg)	52.03 ± 8.81	62.25 ± 7.77	67.04 ± 7.09	66.22 ± 6.42	73.69 ± 9.16
Skeletal muscle mass (kg)	25.78 ± 4.77	31.50 ± 4.02	33.53 ± 3.13	33.74 ± 3.82	37.99 ± 5.30
Body fat (%)	10.48 ± 4.25	10.25 ± 2.64	13.26 ± 6.36	10.43 ± 3.29	9.79 ± 2.11
FMS total score (AU)	14.81 ± 1.94	14.91 ± 1.38	15.50 ± 1.87	14.43 ± 2.17	15.60 ± 1.88
30-m sprint (s)	4.80 ± 0.24	4.47 ± 0.14	4.44 ± 0.16	4.28 ± 0.10	4.21 ± 0.16
CODs with the ball (s)	20.92 ± 0.87	20.29 ± 0.79	20.11 ± 0.83	20.65 ± 1.05	20.09 ± 1.20
CODs without the ball (s)	16.78 ± 0.50	16.01 ± 0.39	15.94 ± 0.37	15.91 ± 0.38	15.73 ± 0.31
Standing long jump (cm)	201.1 ± 19.4	223.6 ± 18.1	228.2 ± 10.8	236.0 ± 20.0	236.5 ± 14.2
Yo-Yo IRTL1 (m)	1152 ± 217	1422 ± 361	1926 ± 450	1720 ± 277	1915 ± 537

Note. Values are mean ± SD. Abbreviations: PHV = peak height velocity; FMS = Functional Movement Screen; Yo-Yo IRTL1 = Yo-Yo Intermittent Recovery Test Level 1; CODs = change-of-direction speed; AU = arbitrary unit.

**Table 2:** Age-category differences in maturity, anthropometric characteristics, body composition, and physical performance outcomes.

Variables	Test	F(df1, df2)	*p*	η^2^	95% CIs
Estimated age at PHV (y)	One-way ANOVA	5.12 (4, 65)	0.001	0.240	[0.051, 0.382]
Maturity offset (y)	One-way ANOVA	45.12 (4, 65)	<0.001	0.735	[0.618, 0.804]
Body height (cm)	Welch ANOVA	5.01 (4, 32.15)	0.003	0.319	[0.117, 0.460]
Body weight (kg)	One-way ANOVA	15.47 (4, 65)	<0.001	0.488	[0.295, 0.609]
Skeletal muscle mass (kg)	One-way ANOVA	16.36 (4, 65)	<0.001	0.502	[0.312, 0.621]
Body fat (%)	Welch ANOVA	0.93 (4, 31.00)	0.460	0.091	[0.000, 0.205]
FMS total score (AU)	One-way ANOVA	0.97 (4, 65)	0.428	0.057	[0.000, 0.149]
30-m sprint (s)	One-way ANOVA	27.97 (4, 65)	<0.001	0.633	[0.478, 0.725]
CODs with the ball (s)	One-way ANOVA	2.08 (4, 65)	0.094	0.113	[0.000, 0.236]
CODs without the ball (s)	One-way ANOVA	16.52 (4, 65)	<0.001	0.504	[0.315, 0.623]
Standing long jump (cm)	One-way ANOVA	11.39 (4, 65)	<0.001	0.412	[0.210, 0.545]
Yo-Yo IRTL1 (m)	Welch ANOVA	16.47 (4, 30.21)	<0.001	0.408	[0.206, 0.541]

Note. Welch ANOVA and Games–Howell post hoc tests were used when the assumption of homogeneity of variance was violated. Abbreviations: η^2^ = eta squared; 95% CIs = 95% confidence intervals; PHV = peak height velocity; FMS = Functional Movement Screen; Yo-Yo IRTL1 = Yo-Yo Intermittent Recovery Test Level 1; CODs = change-of-direction speed; AU = arbitrary unit.

**Table 3:** Multivariable linear regression models examining associations of chronological age and maturity offset with physical performance outcomes.

Outcome	R^2^	Predictor	B [95% CIs]	β	*p*
FMS total score (AU)	0.041	Chronological age	0.489 (−0.087, 1.065)	0.409	0.095
		Maturity offset	−0.482 (−1.144, 0.179)	−0.351	0.150
30-m sprint (s)	0.545	Chronological age	−0.097 (−0.154, −0.040)	−0.561	0.001
		Maturity offset	−0.039 (−0.105, 0.027)	−0.197	0.241
CODs with the ball (s)	0.044	Chronological age	−0.232 (−0.537, 0.073)	−0.366	0.133
		Maturity offset	0.157 (−0.193, 0.507)	0.215	0.374
CODs without the ball (s)	0.355	Chronological age	−0.168 (−0.305, −0.031)	−0.485	0.017
		Maturity offset	−0.049 (−0.206, 0.108)	−0.124	0.534
Standing long jump (cm)	0.387	Chronological age	1.906 (−3.308, 7.120)	0.141	0.468
		Maturity offset	7.709 (1.722, 13.700)	0.496	0.012
Yo-Yo IRTL1 (m)	0.332	Chronological age	218.6 (94.48, 342.7)	0.708	<0.001
		Maturity offset	−56.02 (−198.5, 86.47)	−0.158	0.435

Abbreviations: FMS = Functional Movement Screen; Yo-Yo IRTL1 = Yo-Yo Intermittent Recovery Test Level 1; CODs = change-of-direction speed; 95% CIs = 95% confidence intervals; AU = arbitrary unit; VIF = variance inflation factors. Note. VIF = 4.067 for both predictors in all models. Reported *p*-values are unadjusted; no maturity-offset coefficient remained significant after Holm correction (smallest adjusted *p* = 0.074).

## Data Availability

The data presented are available on request from the corresponding author.

## Abbreviations

The following abbreviations are used in this manuscript:

AU	Arbitrary Unit
CODs	Change-of-direction speed
FMS	Functional Movement Screen
PHV	Peak Height Velocity
Yo-Yo IRTL1	Yo-Yo Intermittent Recovery Test Level 1
